# Medical procedures in children using a conceptual framework that keeps a focus on human dimensions of care – a discussion paper

**DOI:** 10.1080/17482631.2019.1675354

**Published:** 2019-10-17

**Authors:** Katarina Karlsson, Kathleen Galvin, Laura Darcy

**Affiliations:** a Faculty of Caring Science, Work Life and Social Welfare, University of Boras, Boras, Sweden; b School of Health Science, University of Brighton, Brighton, UK

**Keywords:** Young children, medical procedures for children, humanization, dehumanization, suffering

## Abstract

**Purpose:** Children’s perspectives in the context of health service delivery have historically been seen as unimportant. They have been viewed as unintelligent, unable to effectively share or tell of their experiences or fully participate in their care, potentially resulting in a sense of dehumanisation.

**Method:** The present paper illustrates children’s experiences when undergoing medical procedures, using application of the eight dimensions of humanised care theoretical framework.

**Results:** Findings from six published papers were reflectively interrogated to identify implicit findings related to the dimensions of humanised care. These implicit findings show ways of caring for childrenwhichcan lead to enhanced human sensitivity in care or conversely where the dimensions of being human are obscured to greater or lesser degrees and can result in forms of dehumanisation.

**Conclusions:** Inadvertent dehumanising features of practice can be mediated by encouraging the inclusion of children’s own lifeworld perspective and make room for their voices in both care and research. In this way the present well documented power imbalance could be addressed. Adding the value of the theoretical framework highlights areas of need for young children to be cared for as human beings.

Teo is 4 years old and he is afraid. Why doesn’t anyone talk to him? He doesn’t want the needle stick. Why doesn’t anyone understand that? Teo holds tightly onto his t-shirt so no one can take it off over his head. Now, the struggle begins. Pappa and the nurse try to take off Teos t-shirt while talking calmly to him. Teo starts to cry—quietly at first but stronger and louder. “I don’t want the needle” he screams over and over. Pappa and the nurse say he has to and it will only be worse if he carries on like this. Teo is afraid and wonders what they mean about his carry on? He is afraid. He can’t think, it’s difficult to breath and he is crying a lot. The t-shirt is pulled over his head and he takes it and holds it very tightly with both hands in front of him. Pappa lifts Teo up in the air in order to place him lying down on the examination bed. He holds Teos hand with his hands while pushing him down onto the bed. Now another nurse comes in to hold Teo’s other arm. She takes off the anaesthetic cream washes and prepares to insert the intravenous cannula. Teo cannot move. The only things he can do are kick and scream and he does just that. “I don’t want the needle stick, I’m afraid, I want mommy” he repeats several times, crying. It’s more and more difficult to hear what Teo is saying now. A nurse holds his arm while the other sticks him. After a while, Teos stops screaming but he is sweating and exhausted. Sniffling, he whispers after his Mommy for a few more minutes”

## Introduction

The illustration from Teo, above, highlights how easily the child’s dignity as a human being can be assailed in the context of necessary medical procedures in treatment and care. Further, in current practice, the child’s chronological age rather than level of distress often guides procedures (Bray, Snodin, & Carter, 2015).

### Children’s experiences of medical procedures

Several studies show that children, regardless of age, experience needle procedures as painful and intimidating (Kettwich et al., 2007; Salmela, Aronen, & Salanterä, 2009, 2011; Taddio, Aronen, & Salanterä, 2014). If children experience painful medical procedures while not really understanding the purpose, the feeling of fear increases. They may also feel abused afterwards (Salmela et al., 2011). For instance, children’s’ experiences of vaccination have taught us that children experience fear that the vaccination will harm them (Harder, Christensson, & Söderbäck, 2014). The pain experienced increases fear, which in turn increases the pain (McMurtry et al., 2015; Taddio et al., 2009). Children do not get used to pain, rather, the child’s fear decreases if the child becomes familiar with the situation/surroundings (Kortesluoma & Nikkonen, 2004). According to parents, children experience more anxiety and fear than pain. Anxiety and fear reinforce the child’s pain, but if the child understands what happening and can think of something positive and nice, the child’s fear may decrease and therefore also the pain (Cohen, 2008). In addition to pain management, an extended focus on fear reducing interventions is suggested for needle procedures (Hedén, Essen, & Ljungman, 2016).

### Restraint during clinical procedures

Children are frequently held for clinical procedures so that they are completed. Restraining children during medical procedures creates anger, discomfort and causes the child to resist (Snyder, 2004). Younger children and those requiring procedures perceived as urgent are more likely to be held (Bray et al., 2015). There is an overreliance on holding children that raises many ethical and moral issues that appear to be overlooked. The holding of children for clinical interventions and procedures is often uncontested in practice. Distressing procedures such as taking blood tests, administering medication, x-ray, eye drops and nebulizer require children to be held tightly, without analgesia, in current practice (Bray et al., 2015; Brenner, 2013). Healthcare professionals consider it better to restrain the child so that the procedure is implemented (Cummings, 2015; Ives & Melrose, 2010; Söderbäck, 2013) and the pain and discomfort the child is exposed to justified by the fact that adults believe it is in the child’s best interest. The pain and discomfort healthcare professionals conduct against the will of the child is often justified by adults as contributing to what is best for the child (Llyod, Urquhart, Heard, & Kroese, 2008). Research indicates that healthcare professionals’ demonstrate perseverance, in spite of suffering caused, while they simultaneously value being child centred. In order for professionals and parents to pursue a child-centred strategy, the child needs to be calm throughout the procedure—if the child becomes upset then adults agreed that the procedure needed to be completed regardless of the level of restraint required (Bray et al., 2015). Children’s’ lack of cooperation and upset is perceived as a challenge and obstacle to be overcome rather than as a trigger for professionals to question their actions. Many clinical procedures are brief and performed frequently by healthcare professionals creating a context in which the end is always in sight for the adults, in other words, the end justifies the means. They continue regardless, despite evident disquiet about their boundaries of practice being stretched. Furthermore, holding down is described as expected, acceptable and a necessary breach of trust which is often uncontested in practice (Bray et al., 2015; Brenner, 2013). Parents and healthcare professionals’ experience moral distress expressed as uncertainty, guilt and upset when breaching the trusting and protective relationship established with children (Bray et al., 2015). Llyod et al. (2008) have highlighted some of the experiences described by nurses when managing children having invasive medical procedures and shown how these are undertaken in clinical settings. While the results cannot be generalized beyond the specific study setting, there are clear indications about the kind of emotions and concerns that might be experienced by healthcare professionals in these kinds of situations. Participants in the focus groups suggested that the support they received may not be available to nurses managing children outside of paediatric settings (Llyod et al., 2008). Research suggests that even nurses themselves interpret the child’s behaviour as the child being ashamed during the procedure when restraint is used (Ives & Melrose, 2010). This is in contrast to, from a child’s perspective, restraint during medical procedures leads to feelings of fear, anger, confusion and emotional stress (Bray et al., 2015; Brenner, 2013; Coyne & Scott, 2014). Further, lack of clarity regarding when or how to use restraint in paediatric nursing is in direct contrast to international legislation, in addition, children’s rights activists are continuously supporting increased safeguards to protect children and to contribute to improved health-care services for children. According to Bray et al. (2015) restraint “happens all the time”- but a key question here is: Should it? The literature paints a picture of great suffering as a result of everyday medical procedures whereby children can and often do experience a loss of human dignity through for instance, loss of freedom, being rendered passive, forms of objectification, dislocation manifest as disrupted connection with family and loved ones and struggles to find meaning and sense of personal journey when within the “treatment system”, which necessarily prioritizes treatment. However, this necessary focus can easily obscure or even forget the child as a human person altogether. The focus of this present paper is to explore one way of how this might be mediated in caring practices.

### The relevance of a humanizing theoretical framework

The ability to experience making choices and decisions gives personal freedom and a sense of agency. If the possibility of freedom to be and act in a certain way is taken away, then the sense of personhood and therefore personal sense of human dignity can be diminished. Individual choice by children under medical care is rare. Rendering children passive can at the extreme strip them of human dignity, and less extreme forms of passivity can obscure a sense of personal agency to various degrees, at its extreme this can be experienced as dehumanizing (Todres, Galvin, & Holloway, 2009). To be human is to be unique and we all have a sense of our own individuality. De-Emphasizing a person’s uniqueness and individual preferences so that they “fit in”, can result in homogenization. For example, taking on a role as a patient places certain expectations upon them (Todres et al., 2009). Studies have indicated that the ability to make decisions is vitally important for children during medical procedures (Coyne & Kirwan, 2012; Runeson, Hallström, Elander, & Hermerén, 2002). When children participate in decisions concerning their care, it facilitates their ability to cope with their current situation. When children, especially during medical procedures, are taken account of and are listened to, the child’s participation is enhanced (Coyne & Scott, 2014) and by participating, children can then handle their fear more easily (Salmela, Salanterä, & Aronen, 2010a; Salmela, Salanterä, Ruotsalainen, & Aronen, 2010b). A child’s need of control is an important coping strategy in order for them to specifically handle needle care procedures (Ayers, Muller, Mahoney, & Seddon, 2011).

Our uniqueness exists in relation to others and a sense of belonging is fundamental to personal well-being (Borbasi, Galvin, Adams, Todres, & Farrelly, 2013). In separation, we feel separated from our sense of belonging to others. Undergoing healthcare procedures causes disruptions to everyday social connections and we can feel lonely and alienated. Children are understood not only as individuals but in the context of being part of a family. Many studies confirm the importance of having parents present and participating when children are in contact with healthcare (Björk, Nordström, & Hallström, 2006; Runeson et al., 2002; Salmela et al., 2009, 2011, 2010a). Children are afraid of unknown people, which cause feelings of fear and insecurity when children come into contact with care services (Salmela et al., 2011). If children are separated from their parents, everyday social connections are disrupted and they can naturally feel lonely and alienated. In this situation their sense of belonging is potentially diminished. To counteract this situation, studies demonstrate that parental comfort, tenderness and sense of nearness to parents are important actions (Salmela et al., 2010a). Parental comfort, serenity and closeness are specifically described by Salmela et al. (2010a) as important measures for the child’s experiences of sense of security and safety in contact with healthcare and that they, in fact, constitute the child’s coping strategy when on the receiving end of healthcare. When children are afraid of needle stick procedures, parents are important for the child’s experience of feeling safe (Runeson et al., 2002; Björk et al., 2006; Salmela et al., 2009, 2010a, 2011). Research has highlighted that parents should not participate in healthcare procedures in a way that may affect the parent’s ability to act as safe base for the child (McGrath, Forrester, Fox-Young, & Huff, 2002; Pearch, 2005; Schechter et al., 2007). So a sense of personal belonging and connection to family and parents is central and this should not be disrupted through parent’s involvement in medical procedures. To be human is to have a sense of coming from a particular place, where the feeling of being at-home or feeling at home is meaningful. Such a sense of place constitutes the kind of belonging that provides a degree of security, comfort, familiarity; continuity and unreflective ease (Todres et al., 2009). In the healthcare context, there is a need to make sense of one’s surroundings, to facilitate a sense of ”homeliness” in the face of a new environment (Borbasi et al., 2013). Making sense of things, events and experiences is a fundamental human trait. When sense-making is obscured to varying degrees a sense of dislocation and meaninglessness can be experienced. Thoughtful practice helps children make sense of their situation and surroundings when familiar details and routines are lost in changing circumstances linked to care and sense of family belonging is central in this regard.

Living within the fragile limits of human embodiment is to be human. In a reductionist view, there is an overemphasis upon signs and symptoms and the body as separate from a broader concept of being a child. Medical procedures can become the professional focus of care, not the child (Bray et al., 2015; Brenner, 2013). Within a humanizing perspective, a sense of wellbeing as a holistic experience is a positive quality for the child rather than experiencing the situation as *just a body to be fixed*. Thus, focus needs to be redirected to the child’s needs and experiences and not just stay with a reductionist view of the ill child, and signs and symptoms in-order to enhance humanly sensitive care.

Added to the relevance of these dimensions in the care of the ill child, the literature is replete with examples of how children’s own views on health care have historically been seen as inaccessible and unimportant, instead, their parents have been their representatives. They have been viewed as unintelligent and unable to tell of their experiences or participate in care (Coyne, 2006) and this can add to suffering and could be argued can contribute to forms of dehumanization. Further, the decision of how a child’s best interests are served continues to be highly subjective. This is against a backdrop where children have a moral right to have their voice and protests heard and respected and for these to inform judgements of what is in the child’s best interest. Despite this knowledge, the existence of children’s rights (UNCRC, 1989) and the availability of alternatives to holding in needle stick procedures, children are not always respected as active participants in their care, and further avenues to enhance the child’s participation are not always explored. In addition, the policy context is very clear: children’s participation in health care is something that is noted in the Children’s Convention (UNCRC, 1989) and in the Health Care Act (SFS, 1982:763). In order to clarify the patient’s position in Swedish healthcare, the new Swedish Patient Law (SFS, 2014:821) was implemented in the beginning of 2015, with the aim of supporting patient integrity and opportunities for participation in care. In this document, the child’s participation is also noted and in order to get the best information about children we need to place the child and their perspective in the centre and access information from the child her/himself. In order to offer new insight as to how this could be achieved in paediatric practice, and to offer practical directions for this policy and evidence context, children’s experiences when undergoing medical procedures were revisited. Using the eight dimensions of humanization of care, as sensitizing attunement for practices, the humanizing value base demonstrated might encourage practices where the child is meaningfully at the centre of care. In this way, we suggest that the child can be met as a human and how their unique perspective is paramount in humanly sensitive care.

**Figure 1.**
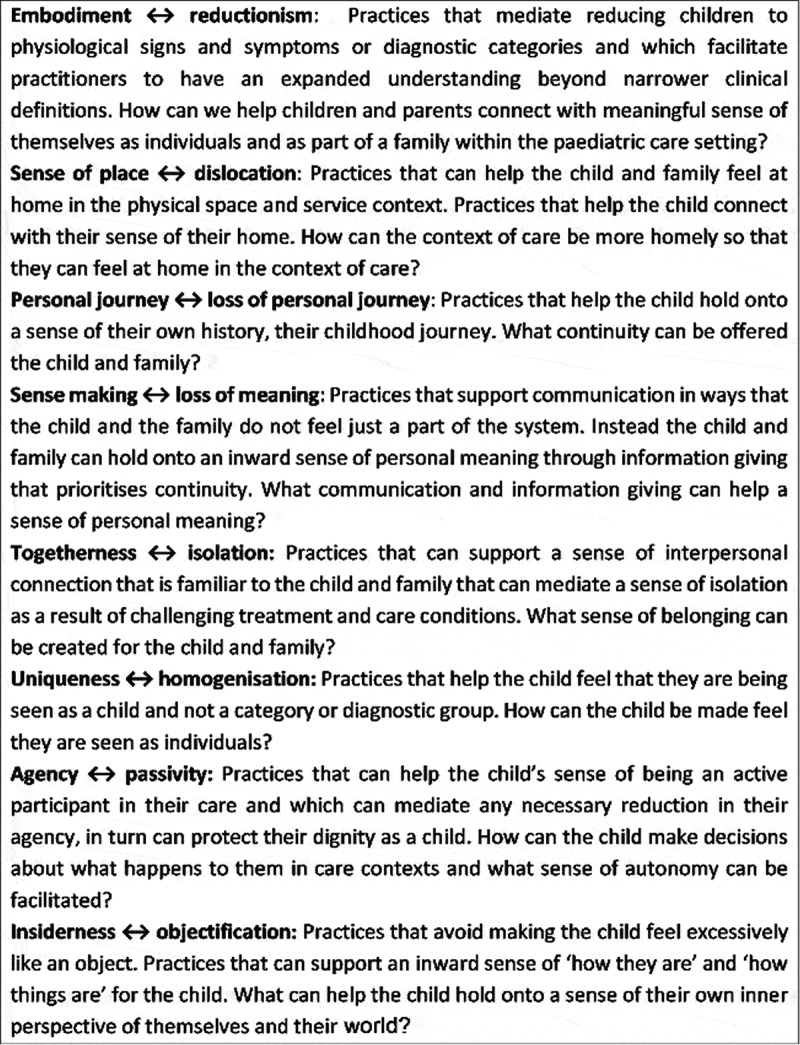
Eight dimensions of what it is to feel human within the paediatric care setting.

We are interested in how a conceptual framework consisting of eight dimensions of what it is to feel human that has been proposed by Todres et al. (2009), can offer a value base for enhancing humanly sensitive care in this context. By illustrating these humanizing dimensions, drawing on existing published research, we hope to offer practical directions to respond to the needs of young children and how they can be cared for as sensitively as possible in the context of necessary and sometimes painful and very distressing treatments. The following figure (Figure 1) summarizes the eight dimensions, each delineate a core feature that can guide what needs to be attended to in order for the child to feel more or less human. Conversely, if the emphasis on one or all these dimensions are obscured or forgotten altogether in care, then there is the possibility of the child feeling less than human in the care context. Each of the eight dimensions within the humanizing care theoretical framework (Todres et al., 2009) are described, with example practices.

### Aim

The aim of this paper was to illustrate children’s experiences when undergoing medical procedures using the eight dimensions of humanization conceptual framework.

## Method

### Design

The present paper draws on the detailed results of six separate studies undertaken in South West Sweden between 2011–2014. It was not our aim to offer a systematic review, rather we aimed to analyse a group of papers that could serve to reveal some new depth about caring for young and ill children during medical procedures. This is because there is not enough reflection on existing qualitative research and how it can offer new insights to guide practice (Todres, Galvin, & Dahlberg, 2014). Our method was to interrogate some essential themes given by a conceptual framework for the human dimensions of care that were implicit in relevant publications that might lead to insights for paediatric practice. Therefore, we specifically sought and selected papers that concerned the child as research participant in South West Sweden in context of care in severe illness. The methodological aim is to take another “step back” from research literature and to reflect on the findings from a group of papers deeply, using the human dimensions of care as a sensitizing reflective context. In this way, we hoped to inform practical directions to mediate the impacts of necessary medical procedures. Eligible papers (Table I) were selected (Noblit & Hare, 1988) because they concerned the young child’s experiences of undergoing medical procedures related to treatment and care as a result of a serious illness, in Sweden. The rationale was to provide rich findings that described experiences from both the unique child’s perspective, in addition to complementary parental insights, that could then be reflected upon in the context of the humanized care theoretical framework. The overarching motivation was to explore the findings in-depth and to use reflection to illuminate new insight and point towards aspects important in care in a discovery-oriented way rather than to offer any summative analysis in a conventional way (Todres et al., 2014). The present study illustrates the usefulness of the humanized care values framework to offer practical directions to enhance care of the child. So we are not offering a systematic review of the literature, but rather a fresh analysis of a group of papers that might reveal something new about practice. In support of our methods, we draw also on Strike and Posner (1983) who have suggested that qualitative synthesis should involve some conceptual innovation (in this case use of a conceptual framework to delineate human dimensions of care) which can involve some re-interpretation of published findings rather than relying only on primary data. Our process also followed the following guiding steps: 1. Deciding what is relevant to our initial interest 2. Reading and reflecting on the selected studies. 3. Determining how each study is related to each or all of the eight human dimensions of care through a reflective process 4. Offering some insights in relation to each theoretical dimension that could be practically useful.

**Table I.** Overview of studies.

Paper	Title	Design	Method	Analysis
1	**The everyday life of the young child shortly after receiving a cancer diagnosis, from both children’s and parents perspectives**. Darcy et al. (2014a)	Explorative descriptive	Interviews at 3–9 weeks post diagnosis	Qualitative Content Analysis
2	**The process of striving for an ordinary, everyday life, in preschool aged children living with cancer, from six months to one year post diagnosis**. Darcy et al. (2014b)	Explorative descriptive	Interviews at six and 12 months post diagnosis	Qualitative content analysis Synergy of data from two time-points
3	**Following young children’s health and functioning in everyday life through their cancer trajectory**. Darcy et al. (2015)	Longitudinal deductive	Interviews and questionnaires at diagnosis, six, 12, 18, 24 and 36 months post diagnosis	Quantitative descriptive statistics
4	**Consequences of needle-related medical procedures: a hermeneutic study with young children (3–7 years)** Karlsson et al. (2016a)	Interpretive	Participant observation and lifeworld interviews	Interpretive lifeworld hermeneutical analysis
5	**Experiencing support during needle-related medical procedures: a hermeneutical study with young children (3–7 years)** Karlsson et al. (2016b)	Interpretive	Participant observation and lifeworld interviews	Interpretive lifeworld hermeneutical analysis
6	**Parent’s perspectives on supporting children during needle related medical procedures** Karlsson, Dalheim Englund, Enskär and Rydström (2014)	Descriptive	Lifeworld interviews	Descriptive phenomenological analysis

### Six selected studies

Children (1–6 years of age) and their parents were interviewed about their experiences of everyday life with cancer in studies 1–3. Data were gathered over a three-year period; shortly after diagnosis, and six, 12, 18, and 36 months after diagnosis. Interviews were analysed qualitatively by content analyses according to Elo and Kyngäs (2008). Parent’s views were welcomed as complementary to child data (Table II).

**Table II.** Characteristics of participants in Studies 1–3.

	3–9 weeks after diagnosis n=13	6 months after diagnosis n=12	12 months after diagnosis n=12	18 months after diagnosis n=12	3 years after diagnosis n=12
**Age of child in years**					
1	3	2	-	-	-
2	3	2	3	2	-
3	2	4	3	2	1
4	4	4	3	4	2
5	1	1	3	3	3
6	-	-	-	1	3
7	-	-	-	-	2
8	-	-	-	-	1
**Diagnosis**					
Leukaemia	9	9	9	9	9
Solid tumours (incl. brain tumours)	4	4	3	3	3
**Undergoing treatment**					
Active	13	12	9	9	1
Follow-up	-	1	3	3	11
**Gender**					
Female	9	9	8	8	8
Male	4	4	4	4	4
**Place of interview**					
Home	5	8	10	9	11
Hospital	8	5	2	3	1
**Length of interview**					
(median in minutes)	77	89	100	77	85
**Child participated in the interview**					
Yes	9	10	10	10	12
No	4	3	2	2	-
**Parents participated in the interview**					
Mother and Father	10	8	8	5	6
Mother only	3	5	4	6	5
Father only	-	-	-	1	1

Studies 4–6 involved children (3–7 years of age) and their parents, with a variety of diagnosis who were observed and interviewed about their experiences of undergoing needle-related medical procedures. Data were gathered qualitatively with reflective lifeworld research (Dahlberg, Dahlberg, & Nyström, 2008) through observations and interviews with children during needle-related medical procedures and analysed with Lifeworld hermeneutics or Phenomenological approaches (Table III).

**Table III.** Characteristics of participants in Studies 4–6.

Demographics of the children (*n* =21)	*n*
Age of children (years) 3 4 5 6 7	4 3 5 6 3
Gender of the child Female Male	11 10
Parents present during the NRMP Mother Father Both mother and father	14 3 4
Type of visit Not scheduled Scheduled	5 16
Diagnosis Allergy Cancer Gastrointestinal and bowel disease Genetic disease Infection Nonspecific Obesity Rheumatoid arthritis Tonsillectomy	3 4 1 1 3 4 1 2 2
Reason for the visit Investigation Infection Treatment (e.g. cancer, RA) Scheduled surgery	8 3 8 2
Type of NRMP Capillary blood sample Venous blood sample Intravenous cannula insertion (IV) Needle insertion into a central venous port Injections into the joints Skin testing for allergies	5 5 3 5 1 2
Pharmacological treatment Inhalation/Sedation with N_2_/O_2_ Topical anaesthesia: EMLA® Topical anaesthesia: Rapydan® (Children received pharmacological aids according to the regular routines established within each unit)	2 13 1
Time for the procedures	*Minutes*
NRMP Interval Mean Median Interviews Interval Mean Median	4–30 11 10 9–60 36 37

### Data analysis of the present study

Based on Todres et al. (2009) eight dimensions of humanized care the authors engaged in a reflective process by reading and rereading findings and excerpts of data from all six papers. Sensitized by the dimensions of the theoretical framework examples of suffering related to care and treatment were identified, discussed in relation to humanized care dimensions and highlighted in the text. The highlighted text was then clustered to form examples of humanization and/or forms of dehumanization that can illustrate the usefulness of the framework as a value base, offering practical directions.

#### Ethical considerations

The Ethical Review Board, Linköping, Sweden gave formal approval for studies leading to papers 1–3, (dnr 2010/343-31) and The Ethical Review Board, Gothenburg, Sweden gave formal approval for studies leading to papers 4–6, (dnr 724-10, T099-12). Guidelines for Ethical Evaluation of Medical Research involving Human Subjects were followed (SFS, 2003:460). Signed informed consent was gathered from parents at the start of the study and the children that could gave their verbal assent to participate. The children and their parents were, at each time point, assured of confidentiality and that they could withdraw from the study whenever they wanted, without the decision affecting any future care.

## Findings

The reflective process resulted in contextualized descriptions of care of the child with illness, with pointers to forms of humanization and dehumanization as a continuum. Using the theoretical framework as a sensitizing attunement a spectrum of positive humanizing possibilities, through to negative dehumanizing conditions and contexts can be suggested. From a lifeworld perspective each of the eight dimensions is interwined, so they overlap, but we draw them out as distinct dimensions for the purposes of the paper.

### Insiderness ↔ objectification

During medical procedures, focus needs to be directed on the child, specifically their inner world, their needs and their experiences so that there is an emphasis on a sense of insiderness. Children’s opinions are seldom requested, their inner experiences are not explored or taken account of which may lead to a kind of objectification. This could be exacerbated when fear is the most salient feeling children experiences during different procedures (Darcy, Knutsson, Huus, & Enskär, 2014a; Karlsson, Rydström, Nyström, Enskär, & Dalheim Englund, 2016a). Several of the papers provided evidence (Darcy, Björk, Enskär, & Knutsson, 2014b; Darcy, Knutsson, Enskär, Granlund, & Björk, 2015; Darcy et al., 2014a) that incidents of objectification are common and occur for example, when young children were diagnosed with cancer (or long-term illness). At diagnosis and start of treatment parental and healthcare professionals focus was solely on the illness and not on the child, resulting in an obscuring of insiderness and potentially contributing to objectification. Practical directions can include not focusing on the diagnosis or categorization of illness and treatment pathway exclusively but keeping a focus on the child, their connections with time, space, others and their body.

Ways in which professionals can lean towards insiderness include healthcare professionals striving to be clear about their own boundaries when starting or continuing a procedure when a child is communicating distress. This includes encouraging preparation and engagement activities with children and parents before, during and after medical procedures. This can be done by letting the child be the centre of attention and invite participation by encouraging the child in connection with all aspects of the procedures. Another way is to help the child by distraction strategies but to be aware that not all children want to be distracted for example: Nurse: *Do you want to watch or read a book? The child looks at what the nurse is doing. A book is put in front of the child’s face. Child: I want to see. The child looks upset and quickly takes the book away with one hand* [Observation, six-year-old girl, for example, from Karlsson, Dalheim Englund, Enskär, Nyström, and Rydström (2016b)]. Therefore, a sense of insiderness can be connected with when the child feels that adults make effort to make the procedure convenient and comfortable: *Nurse: Are you lying comfortably now? Child: No. Nurse: You’ve got to move up a little with your bottom. The nurse fixes the pillow and raises the child’s head in the bed. The child settles and begins to read a book* [Observation, six-year-old girl].

### Agency ↔ passivity

Being treated with respect and agency seem to be closely related. Health professionals can help the child achieve a sense of agency. This can be achieved by including children’s own view, decision, actively taking part, no matter how small, maximizing choices and offering all possibilities to be participatory. Ultimately this helped the child to control their own situation during medical procedures. Children interacted well with nurses who actively built a relationship with and made the child part of the procedure in the studies by Darcy et al. (2014a) and Karlsson, Englund, Enskär and Rydström (2014). This behaviour may go some way to rebalance the power imbalances that may occur sometimes inadvertently in paediatric care.

Conversely, the use of restraint during different procedures still happens and negatively affects the child’s sense of agency and their sense of their ability to achieve agency. Restraint evokes feelings of loss of control, panic and powerlessness in addition to rendering the child passive. Passivity can commonly occur when the child and their body are exposed to internal and external forces in care. For example, evidence from Darcy et al. (2014a) and Karlsson et al. (2016a) paper suggests that adults forced the child to do things against their will: *The child is held down by the parent while the nurse inserts the needle. The child is sweating, kicking, and screaming; hitting the nurse in the stomach; crying and trying to get away* [Observation, five-year-old boy]. Further, if the child is rendered passive with no agency so that the child fails to control fear that occurs during medical procedures, feelings of shame may ensue. Feeling ashamed may manifest as a kind of shyness; as in the following example: *The child buries her face in her mother’s neck, looking down at the floor. It seems like she is trying to hide her face. She looks ashamed* [Observation seven-year-old girl]. Therefore, a sense of limited agency can negatively influence the child’s sense of themselves in very extreme ways.

Health professionals included parents in aspects of care and as assistants to painful procedures that left children feeling unprotected. Here a sense of belonging was obscured with negative impacts and risked making the child feel alone. The child expressed having no control over his/her own body and not having parents as protectors during painful procedures left the child abandoned, powerless and contributed to suffering: *Those first few days after we had to hold her down she didn’t want anything to do with us. We had to work hard to build up his trust again* [Interview, mother of a two-year-old boy]. It is therefore important that children are not restrained by their parents or close individuals that give them a sense of belonging (Darcy et al., 2014a).

### Uniqueness ↔ homogenization

Children are sometimes viewed as their diagnosis alone, which increases the risk of homogenization. An example of this is when health professionals focus on the child´s diagnosis to such an extent that children with serious long-term illness are expected to be used to medical procedures and accepting of them. Subsequently, they are expected to already have understandings of procedures, rather than a focus and not on whether the child recently been through the procedure: For example, *They treated her as though she still was used to it … but she wasn´t … maybe she needed at little more time for preparation, information”* [Interview, mother of a four- year-old girl] (Karlsson, Englund, Enskär, & Rydström, 2014).

Children also placed importance on how health professionals entered their room and greeted the child directly. An emphasis on uniqueness can be achieved by giving the child the opportunity to tell everyone how they feel, to take the time to sit, talk and show interest in them as a person. This can also help the child maintain their personal sense of control during procedures, can counteract homogenization, i.e., as a child with long-term illness who should be used to the system and instead recognize the child as unique individuals in an ongoing way: *It´s important how staff treat the child, how they approach him before they do anything, it makes a difference to how he takes it … if they just takes that extra minute and just talk to him and maybe ask him if he wants to help* [Interview, father of a four-year-old boy] (Darcy et al., 2014a)

Likewise, when health professionals make children their central concern and connect with them on the child´s own terms stating, for example, *You’re doing this really well … You are the best in the world*. Health professionals can also recognize children as unique individuals by paying attention to the child´s difficult feelings by saying *It’s okay to be sad and cry*. Acknowledging the child’s suffering is important in mediating sense of homogenization and requirement “to fit into the system” even if the professional cannot change the treatment requirement or how the child is feeling.

### Togetherness ↔ isolation

Children primarily seek security from parents but they also need the whole family to be present in hospital which gave them a sense of togetherness manifest as a kind of security: *It was very important to her that we all stay here together in the hospital, very important … she checks that everyone is here* [Interview, father of a two-year-old girl] (Darcy et al., 2014a).

Children who have established a relationship with health professionals can potentially experience a sense of togetherness and can more easily entrust him/herself and let the health professionals perform the procedure. Likewise, when children meet other children who are in the same situation, both being ill and in need of needle procedures, feelings of togetherness may be strengthened.

To encourage this sense of togetherness, feelings of loneliness and isolation must not be allowed to develop. Isolation occurs when children sense separation from parents, siblings and friends: *I always played with my best friend before, now we never play* [Interview, four-year-old girl]. Isolation may also occur if the parents are worried and sad about the child’s illness or medical procedure (Karlsson et al., 2016a). This can lead to feelings of sadness and loneliness, which this quotation exemplifies: During the interview, the child asked. *Mom, why did you cry before?* [Interview, six-year-old girl]. Feelings of being abandoned may occur if the child feels that the parents are not protecting them: Mom: *Just do it [the needle stick]. She brings the boy’s hand towards the nurse. The child begins to cry, trying to hide his hand. Mom: Just do it. Child: No. He looks sad and disappointed* [Observation, four-year-old boy] (Karlsson et al., 2016a).

### Meaning-making ↔ loss of meaning

Thoughtful practice helps children make sense of their situation and surroundings when familiar details and routines are lost in changing life circumstances. For meaning-making to occur the child needs to be allowed to participate and in order to do so, they need to know what is going to happen. In order to gain this specific knowledge the child is often curious (Darcy et al., 2015; Karlsson et al., 2016b). They ask questions about the procedure and they can be invited to tell the nurse how the procedure should be performed: For example, the nurse is about to pull the needle out from the central venous port. Child: *But, we must also have a dry compress?* [Observation, seven-year-old girl]. Another example of emphasizing meaning-making is when choosing words and phrases, finding the right fit for the specific moment: *A child says while the anaesthetic patch is removed: ‘t’s as slow as a snail when it slides*.[Observation, five-year-old boy] (Karlsson et al., 2016a). Another example of how metaphors can be used that can help the child achieve meaning is: *I think it’s like a small aircraft … You can refuel … You can say “the airplane has crashed”* [if the intravenous cannula insertion fails] [Observation, six -year-old girl].

Some of the studies revealed how preparations are a window of opportunity to being child centred and achieving meaning-making (Darcy et al., 2014b, 2014a; Karlsson et al., 2014): Child: *Look, there is a [mechanical] spring [a capillary device]. First, I did it like this, I pressed like this and then a needle was pushed out. Can you only press it once? The child asked this question while looking at the nurse* [Interview, six-year-old girl]. Preparations and information are also important when the child experience difficulties related to having an illness in order to gain meaning-making: *Have to take the tests and stuff … because that’s good … so that I´ll get better and that … so that those mean cells won´t … I don´t want them in my body. Stupid cells! Get out!*[Interview, four-year-old girl] (Darcy et al., 2014b).

A sensitive nurse used toys and props to make the child feel safe and secure during procedures. Security and a sense of control were connected to information and participation in care.

The need to play was important, especially hospital play: *Look! I’m going to take a blood sample from my doll … .but my doll is sad and doesn’t want to go to the doctor* [Interview, three-year-old girl].

Appropriate information and preparation for the child is sometimes lacking and this can result in loss of meaning: for example: Angry, upset child shouting at the doll: *You must be strong! If you are screaming and crying you are not strong* [Observation, five-year-old boy]. Alternatively, if the information that the child is receiving is not adapted specifically to them as individuals, there is a risk that the negative imagination can take over. This may lead to terrifying procedures and difficulties understanding them, here loss of meaning emerges as a kind of suffering of being caught in a negative cycle.

Further, lack of continuity in care meant that health professionals and children often did not get to know each other well enough to use these kinds of strategies in order to prevent the child feeling powerless, when having a medical procedure as the healthcare professionals were not able to reach towards the child in a familiar and homely way.

### Personal journey ↔ loss of personal journey

Personal history and stories help people connect with their sense of self—who they are, not just the how they are. Helping children to remember their everyday life and find connection with it. For example, recognizing and responding when they have thought about things from their everyday life: *Do they think about me at daycare? Do they still call out my name to see if I’m there* [Interview, five-year-old girl].

The studies by Darcy et al. (2014a) and Darcy et al. (2015) indicated that the suddenness of diagnosis and start of treatment led to feelings of loss of personal journey and at its extreme to feelings of being abused by the treatment. Everyday life was spent at hospital or at home waiting to go back to hospital and waiting for and having to endure and face different procedures. The child life was suddenly unfamiliar and utterly changed with feelings of strangeness, powerlessness and loneliness. The child’s everyday life functioning is affected resulting in loss of the child’s personal journey. This is also intertwined with togetherness and isolation and loss of meaning.

Loss of personal journey is also evident when health professionals do not take into account how previous life events have affected the child. For example, when the health professionals focus on the present as a snapshot and disregard previous experiences that have impacted the child and which are significant to them in their journey. Children need help to remember and make connection with their everyday life, their past is as important as the now and their future. Helping the child hold onto their sense of personal journey, who they are, not how they are is an important emphasis in care.

### Sense of place ↔ dislocation

To be human is to come from a particular place, where the feeling of at-homeness is meaningful. Parental presence is very important to the child in order to feel a sense of place. Children wanted the physical closeness and security that a parent can provide and strongly expressed the need for parents to be a safe haven during procedures: Child: *It should be said quietly [the conversation] and hold hands. Interviewer: Who should hold hands? Child: Mom or dad* [Observation, seven-year-old girl].

Dislocation, on the other hand, a sense of homelessness is experienced at home, at the hospital and at preschool/school where unfamiliar disruption obscures the sense of being at home in body, in place, in mood or with others: *She is often sad (at preschool), she’s not able to run or climb like the others and that makes her so sad* [Interview, mother of a five-year-old girl]. Experiences of dislocation and alien contexts are described when the child does not understand why a procedure must be performed, or don’t understand or recognize the surrounding environment or the people involved and this might also bring with it a sense of panic: *… and she screams “help me mummy, help me” … and then we’re on the other side helping the baddies … it feels very wrong, somewhere in all of this it’s in our arms she needs to climb up in when she needs comfort and safety* [Interview, mother of a four-year-old girl]. Dislocation may also occur when the child describes feelings of being disrupted in everyday activities related to their illness and where changes have occurred: *Now I get help from my Mummy to do pretty much everything (before) I did it all myself* [Interview, four-year-old girl] (Darcy et al., 2014a). Any practices that familiarize the child to what is going to happen, who is involved, presence of family and parents, relationship building with key health professional to avoid an emphasis on care through “strangers” can potentially help mediate disruption to sense of place. A sense of being at home in body and not feeling alien in the body is an important aspect of care for the child also and we come to this in considering embodiment and the erosion of a sense of being at home in the body through practices that emphasize a reductionist view of the child.

### Embodiment ↔ reductionist view

A Reductionist view is seen when medical procedures, treatment, signs and symptoms become the professional emphasis and focus of care, with no room for a focus on the child as a person. The need for competent healthcare professionals with a caring approach who could hold onto a view of the child at the same time dealing with medical reductionist emphasis was reported in the studies by Darcy et al. (2015) and Karlsson et al. (2014). In these studies, the children expressed a desire to be treated with respect by being seen *as a child and a person*, by adults. This can be evident in health care when, for example, professionals appear to be unable to see beyond the disease or the sum of tasks to the person behind the disease. Parents also described that healthcare professionals do not have the time to put the child as the focus. This resulted in healthcare professionals’ tendency to “just do”, to “just get to it”, to perform the procedure without involving or informing the child at all. Children liked to be asked permission to lift their t-shirt for examination or invited to actively participate in their care: *It’s also very important how they treat him, you know? How they approach him before doing anything … its makes a big difference to his reaction … that the staff take that extra minute and just talk to him and ask him if he maybe wants to help …* [Interview, mother of a three-year-old boy]. This may lead to reductionist view. Illness and treatment often changed children’s bodies and resulted in new embodiments, this was learning about and coming to terms with a new body that had been through a lot (Darcy et al. (2015): *I have thousands of scars now … one here, one here, one here see? No one else in my class has these* [Interview, seven-year-old girl]. So any practices that involve a slowing down, meeting the child as a person, and seeing the child behind the illness can potentially mediate a reductionist view.

## Discussion

The study process has revealed that there are many examples of everyday practice in caring for young children who are very ill, where sense of embodiment, sense of place, personal journey, meaning–making, togetherness and uniqueness can be attended to in practical ways. More specifically, there are aspects of medical procedures that can easily assail these human dimensions of care and a sensitivity and attunement to these aspects seems vitally important in guiding ethical caring practice. Further, existing literature relevant to paediatric nursing adds to this context. Parents can make the child feel ”more at home” can reinforce a sense of place and belonging when in hospital. Evidence suggests that both children and parents strongly express the need to have parents act as a refuge—being with a parent as a place of safety and comfort the child can retreat to. This is a sense of belonging and metaphorical homeplace.

When the child feels fear and wants to escape the needle procedure, this is usually not allowed by adults. Parents help constrain the child and take part in painful and unpleasant procedures and treatments and a sense of dislocation may obscure feelings of belonging. With this in mind, it is notable that approximately 63% of younger children develop needle phobia after experiences of needle procedures that have not been well managed (McMurtry et al., 2015; Taddio et al., 2012). This can affect children through childhood and into adulthood (Jenkins, 2014), create fear of needle procedures and care in general—20% to 50% in adolescents and 20% to 30% in younger adults (McLenon & Rogers, 2019). Research shows that children with intellectual disability experience more fear and pain that typically developing children during needle procedure (Pascolo et al., 2018), which increases the risk for negative impacts on the child that are long-lasting.

The role of assistant to health-care professionals, assumed by many parents in caring for young children with cancer, has been previously challenged (Björk et al., 2006; Kästel, Enskär, & Björk, 2011). Furthermore, if the child feels like a stranger in daily life related to having an illness or medical procedure dislocation may occur. To be given the opportunity to participate in their own care instead of just being present helps the child in different challenging life situations (Imms et al., 2017). Strategies for collaboration and role definition for parents and health-care professionals need to be reassessed.

If the child cannot control fear during medical procedures and panic occurs, the child is likely to be exposed to restraint and risk of experience passivity. This is in line with the results of Leibring and Anderzén-Carlsson (2019). This shows how important it is to help children manage their fear of needle procedures in order for Agency to appear, or as Hedén et al. (2016) highlight an extended focus on fear-reducing intervention is required. To be given the opportunity to participate in their own care instead of just being present helps the child in different challenging life situations (Imms et al., 2017) and facilitates agency. In the study by Leibring and Anderzén-Carlsson children, aged 5–9 years, various strategies for coping with fear in connection with needle procedures are described as preferred coping strategies such as being given permission to scream out loud and the comfort given by cuddly toys.

Restraint during medical procedures can also be understood on the basis of how Arman (2015) combines caring actions with ethical actions. Caring is about gentle hands ethically caring for a person who is in a vulnerable situation. The caring act should thus be perceived as comforting by the child and lead to a feeling of well-being. Comfort can be difficult to achieve if the child is held down or forced to participate. Without an ethical approach, care can be perceived as instrumental for the child leading to passivity, as described by Todres et al. (2009). It is quite likely that children being restrained also experience what Eriksson (1994) defines as caring suffering.

When restraint is used during painful medical procedures ensuing in a panicked child, it is reasonable to assume that the understanding between child and adult is insufficient. This can lead to a difficult situation for all. A protesting child is highly unlikely to have consented to the implementation of the painful medical procedure. In such scenarios, Nurses, contest the UN Convention (UNCRC, 1989), the Swedish Health Care Act (SFS, 1982:763), ICN’s Code of Ethics (International Council of Nurses, 2012) and the new Patient Act (SFS, 2014:821).

Papers that form the basis of this study (Darcy et al., 2014b, 2015, 2014a; Karlsson et al., 2016b, 2014, 2016a), show the importance for a child to trust adults during medical procedures. Children and adults need to be in connection with others in order to experience togetherness and belonging (Todres et al., 2009). Additionally, Brady (2009) found that for children (7–12 years), trustworthiness is an important quality for nurses in order to be perceived as a ‘good ‘nurse. According to Løgstrup (2009), trust is the basis of an ethical caring relationship, when two people meet, there is the hope and expectation that the other person wishes them well. When the child’s expectation of trust and safety in the adult is met with restraint, it is reasonable to assume that the child feels rejected and the imbalance in the adult’s possession of power becomes clear to the child, they become alienated from their lifeworld. Thoughts presented by Arman (2015) describe that a worthy way of meeting people is by confirming the actual and absolute value of them that can mediate such disruption: To “meet” the child in a way that enables a feeling of being confirmed and not to be offended. To summarize:

- Children need access to parents for refuge and safety—a sense of home and belonging.
- Healthcare professionals and parents need to redefine their roles in collaborative care.
- Children need to be participatory in their own care.
- Fear-reducing interventions could help children manage medical procedures that bring with them pain and fear.
- Children need to be able to fully trust adults during medical procedures.
- Restraint is never acceptable from the child’s perspective.

Such ethically sensitive directions of care are also underscored by some methodological issues of importance when conducting research with younger children (rather than on younger children) and these centrally relate to difficulties in that adults do not see the world the same way as children do. It can also be difficult to linguistically understand each other (Punch, 2002). This observation does not justify relying on proxy studies (Taddio et al., 2014) because, for example, parents underestimate their child’s fear compared to the child’s own self-report. Therefore, paediatric studies are more credible if they make use of authentic child citations (Sandelowski, 1994) and we go further to say that research should go further, the depths and details of child’s lifeworld require illumination. Additionally, Brenner’s (2007) study highlights the need for paediatric nurses and allied health-care professionals to explore sensitive topics head-on. By failing to address sensitive and controversial issues, it could be argued that researchers in child health are ignoring an extraordinarily stressful event in paediatric health care.

## Conclusion

We have aimed to show how often necessary treatment and specific medical procedures can easily assail a child’s sense of feeling human. Dehumanization occurs when one or more of the eight humanizing dimensions of care are obscured to a significant degree. It is apparent from existing qualitative studies that forms of dehumanization can occur in this practice context and extreme examples of, for instance, loss of agency and dislocation are common. Our findings consistently suggest that clouding of what it is to be human can be mediated by some simple practical approaches, such as encouraging the inclusion of children’s own perspectives in care processes. This could involve giving children a choice in the ways in which medical procedures are carried out, always asking about their preferences and actively helping them to prepare. It could also involve identifying and using children’s own coping strategies, such as allowing them to scream and react as well as always protecting the parent’s role as one of homely comfort and security can all enhance practice. Children require assistance in making sense of healthcare situations through play and preparation, encouragement to participate in care and with full access to family and friends. These can constitute directions to enhance human sensitivity in children’s care and can contribute to a greater sense of dignity when on the receiving end of paediatric care. More specifically, the insights of children who have been held should be explored in further research. It is necessary for children, parents and professionals to work together in an informed way to investigate if there is a more human way to perform medical procedures. Young children are competent and research which has given young vulnerable children a voice can also highlight the importance and possibility of always caring humanly for them.

Making use of the humanizing theoretical framework consisting of eight dimensions of humanization can provide a guiding value base that is needed if young children are to be cared for as human beings in every aspect of their treatment. This is particularly important as Sweden prepares for the introduction of the UNCRC as law in 2020 which will require children’s active participation in their care. Application of the theory could offer an attunement and framework of values to help services respond meaningfully to children’s needs in ethically sensitive ways. Children have a moral right to have their voice and protests heard and respected and for these to inform judgements of what is in the child’s best interest. The studies where children are participants that are available overwhelmingly suggest that ethical caring for children in their vulnerable state in paediatric settings requires gentle hands that give comfort. This is difficult to achieve if children are unknowing, uninvolved and forced to participate in medical procedures that they don’t understand. Feelings of rejection and lack of safety have been amply illustrated and can potentially be counteracted by meeting the child as human with a unique perspective, and, using the application of a theory that points to the human dimensions of care can offer a productive value base to guide practice in response to these ethical and policy requirements.
